# Meteorological Observations

**Published:** 1860

**Authors:** J. G. Westmoreland

**Affiliations:** Smithsonian Institution, Nov., 1859, at Atlanta, Ga.


### METEOROLOGICAL OBSERVATIONS,

Taken by J. G. Westmoreland, M. D., for the Smithsonian Institution, Nov., 1859, at Atlanta, Ga., Latitude 33 deg. 45 min.—Longitude 7 deg. 30 min.—Height above the Sea, 1050 feet.

| Days of Month | BAROMETER. | | | THERMOMETER. | | | RAIN AND SNOW. | | CLOUDS. | | | WIND. | | |
|---|---|---|---|---|---|---|---|---|---|---|---|---|---|---|
| | 7 A.M. | 2 P.M. | 9 P.M. | 7 A.M. | 2 P.M. | 9 P.M. | Amt. Rn | In. Sn'w | 7 A.M. | 2 P.M. | 9 P.M. | 7 A.M. | 2 P.M. | 9 P.M. |
| 1 | 30. | 30.05 | 30.05 | 34 | 56 | 42 | | | 0 | 0 | 0 | N W | N W | N W |
| 2 | 30.05 | 30.20 | 30.25 | 38 | 60 | 58 | | | 0 | 0 | 0 | N W | N W | N W |
| 3 | 30.30 | 30.40 | 30.35 | 42 | 68 | 58 | | | 0 | 0 | 0 | N W | N W | N W |
| 4 | 30.33 | 30.35 | 30.30 | 48 | 68 | 58 | | | 0 | 0 | 0 | N W | N W | N W |
| 5 | 30.25 | 30.30 | 30.22 | 48 | 68 | 60 | | | 0 | 0 | 0 | N W | N W | N W |
| 6 | 30.15 | 30.22 | 30.15 | 48 | 68 | 60 | | | 0 | 0 | 0 | E | E | E |
| 7 | 30.15 | 30.20 | 30.15 | 48 | 68 | 58 | | | 0 | 0 | 0 | E | E | E |
| 8 | 30.15 | 30.15 | 30.10 | 50 | 70 | 62 | | | 0 | 0 | 0 | E | E | E |
| 9 | 30.00 | 30.05 | 30.00 | 50 | 70 | 50 | | | 0 | 0 | 0 | S E | S E | S E |
| 10 | 29.90 | 29.92 | 29.85 | 48 | 70 | 62 | | | 0 | 5 | 0 | S E | S | S |
| 11 | 29.85 | 29.95 | 29.90 | 50 | 72 | 68 | | | 5 | 5 | 5 | S | S | S |
| 12 | 29.90 | 29.95 | 29.80 | 54 | 72 | 62 | | | 0 | 0 | 0 | S.E | S E | S |
| 13 | 29.78 | 29.88 | 29.95 | 32 | 36 | 30 | | | 5 | 0 | 0 | N W | N W | N W |
| 14 | 30.00 | 30.15 | 30.12 | 24 | 48 | 40 | | | 0 | 0 | 0 | N W | N W | N W |
| 15 | 30.10 | 30.15 | 30.15 | 32 | 52 | 48 | | | 0 | 0 | 0 | N W | N W | N W |
| 16 | 30.15 | 30.20 | 30.12 | 38 | 58 | 50 | | | 0 | 0 | 0 | N W | N E | E |
| 17 | 30.15 | 30.17 | 30.10 | 44 | 62 | 60 | | | 5 | 5 | 5 | E | E | E |
| 18 | 29.70 | 29.63 | 29.75 | 52 | 58 | 48 | 1.00 | | 10 | 5 | 5 | E | W | W |
| 19 | 30.00 | 30. | 30.00 | 55 | 45 | 40 | | | 0 | 0 | 0 | S W | W | W |
| 20 | 30.00 | 30.07 | 30.05 | 40 | 57 | 49 | | | 0 | 0 | 0 | S | S | E |
| 21 | 29.90 | 29.92 | 29.95 | 50 | 55 | 52 | 1.00 | | 10 | 10 | 5 | E | E | S |
| 22 | 30.10 | 30.15 | 30.20 | 51 | 65 | 68 | | | 5 | 0 | 0 | S | S | W |
| 23 | 30.22 | 30.35 | 30.30 | 48 | 68 | 58 | | | 0 | 0 | 0 | W | W | W |
| 24 | 30.30 | 30.35 | 30.30 | 46 | 66 | 56 | | | 0 | 0 | 0 | W | W | N E |
| 25 | 30.22 | 30.20 | 30.15 | 46 | 62 | 60 | | | 5 | 5 | 5 | E | E | E |
| 26 | 30.10 | 30.07 | 30.04 | 64 | 61 | 60 | 12 | | 10 | 10 | 10 | S | S | S W |
| 27 | 30.00 | 29.95 | 29.95 | 61 | 61 | 60 | 1.25 | | 10 | 10 | 10 | S | S | S |
| 28 | 30.00 | 30.05 | 30.07 | 58 | 62 | 51 | 50 | | 5 | 0 | 0 | S W | W | W |
| 29 | 30.07 | 30.17 | 30.15 | 40 | 58 | 50 | | | 0 | 0 | 5 | N | N | S W |
| 30 | 30.15 | 30.20 | 30.17 | 48 | 61 | 58 | | | 5 | 5 | 0 | E | S W | S |

Explanation of Table.—0 Signifies Entire Clearness—5 Half Cloudy—10 Entire Clearness.

